# Structural characterization of twisted gastrulation provides insights into opposing functions on the BMP signalling pathway

**DOI:** 10.1016/j.matbio.2016.01.019

**Published:** 2016-09

**Authors:** Helen Troilo, Anne L. Barrett, Alexandra V. Zuk, Michael P. Lockhart-Cairns, Alexander P. Wohl, Christopher P. Bayley, Rana Dajani, Richard B. Tunnicliffe, Lewis Green, Thomas A. Jowitt, Gerhard Sengle, Clair Baldock

**Affiliations:** a Wellcome Trust Centre for Cell-Matrix Research, Faculty of Life Sciences, University of Manchester, M139PT, UK; b Center for Biochemistry, Medical Faculty, University of Cologne, Cologne, Germany; c Beamline B21, Diamond Light Source, Harwell Science & Innovation Campus, Didcot, Oxfordshire, UK; d Center for Molecular Medicine Cologne (CMMC), University of Cologne, Cologne, Germany

**Keywords:** Tsg, twisted gastrulation, BMP, bone morphogenetic protein, mTld, mammalian tolloid, Rg, radius of gyration, SAXS, small-angle X-ray scattering, AUC, analytical ultracentrifugation, MALS, multi-angle light scattering, vWC, von Willebrand Factor C, SPR, surface plasmon resonance, MST, microscale thermophoresis, Twisted gastrulation, BMP signalling, Chordin, Tolloid proteinase

## Abstract

Twisted gastrulation (Tsg) and chordin are secreted glycoproteins that function together as BMP (bone morphogenetic protein) antagonists to regulate BMP growth factor signalling. Chordin binds to BMPs, preventing them from interacting with their receptors and Tsg is known to strengthen this inhibitory complex. Tsg also acts as a BMP agonist by promoting cleavage of chordin by tolloid-family proteinases. Here we explore the structural mechanism through which Tsg exerts this dual activity. We have characterized the nanoscale structure of human Tsg using in-solution biomolecular analysis and show that Tsg is a globular monomer with a flattened cross shape. Tsg has a high proportion of N-linked glycans, in relation to its molecular weight, which supports a role in solubilising BMPs. Tsg binds with high affinity to the C-terminal region of chordin and was also able to inhibit BMP-7 signalling directly but did not have an effect on BMP-4 signalling. Although both Tsg and mammalian tolloid are involved in chordin cleavage, no interaction could be detected between them using surface plasmon resonance. Together these data suggest that Tsg functions as a BMP-agonist by inducing conformational change in chordin making it more susceptible to tolloid cleavage and as a BMP-antagonist either independently or *via* a chordin-mediated mechanism. Following single cleavage of chordin by tolloids, Tsg continues to strengthen the inhibitory complex, supporting a role for partially cleaved chordin in BMP regulation.

## Introduction

Bone morphogenetic proteins (BMPs) are secreted growth factors of the transforming growth factor-β family. They are essential for early embryonic patterning, most notably in forming the morphogen gradient controlling dorsal ventral patterning [1]. They also have roles in adult tissues including in muscle maintenance [2] and fracture healing [3]. During dorsoventral gradient formation, chordin is the primary BMP regulator in many organisms including *Xenopus*[4] and zebrafish [5]. Chordin antagonizes signalling by binding to BMPs and preventing them from interacting with their cell surface receptors. This regulation is modulated by the small secreted glycoprotein twisted gastrulation (Tsg) which can form a ternary complex with chordin and BMP [6], [7], [8]. Tsg increases the effectiveness of chordin as a BMP antagonist [6], either by facilitating the interaction between chordin and BMP or by blocking additional BMP receptor binding sites. It is also thought that Tsg stabilizes BMPs in the extracellular space and that N-linked glycosylation of Tsg has an important role in facilitating its interaction with BMPs [9].

BMP inhibition is relieved by cleavage of chordin by tolloid family metalloproteinases [10], removing a BMP-binding von Willebrand Factor C (vWC) homology domain from each terminus [11]. The individual vWC domains have greatly reduced bioactivity *in vivo* and this cleavage has been shown to be an essential regulatory mechanism in patterning. Cleavage at each site is an independent event and fragments lacking both the N- and C-termini have been shown to exist *in vivo* at significant levels [12]. Partially cleaved chordin has been shown to be as effective as full length chordin at BMP inhibition with some gain of function when cleavage occurs at the C-terminal site [13]. Tsg increases the rate of chordin cleavage by tolloids [6] and competes with the residual fragments for BMP binding [14], as well as increasing their rate of degradation *in vivo*[15]. Since Tsg also strengthens the chordin-BMP inhibitory complex, it therefore has a dual effect on BMP signalling [15]. In most species the net effect is that Tsg functions as a BMP agonist but there are exceptions to this, which are thought to be based on endogenous tolloid concentration [16].

The effects of Tsg are well documented but less is known about its mechanism of interaction with tolloids and chordin. One possibility is that Tsg functions as a scaffolding molecule between tolloid and chordin (in a similar manner to Olfactomedin1 in *Xenopus*[17]) and that it increases the cleavage rate in this way. However data suggest that its mechanism of action may involve inducing conformational change in chordin. For example, mouse chordin acquires an additional tolloid cleavage site in the presence of Tsg [6], suggesting that a cryptic site may be exposed following Tsg binding. This would also explain why Tsg has not been shown to exert a broader influence on tolloid cleavage of other substrates in the matrix.

**Fig. 1:**
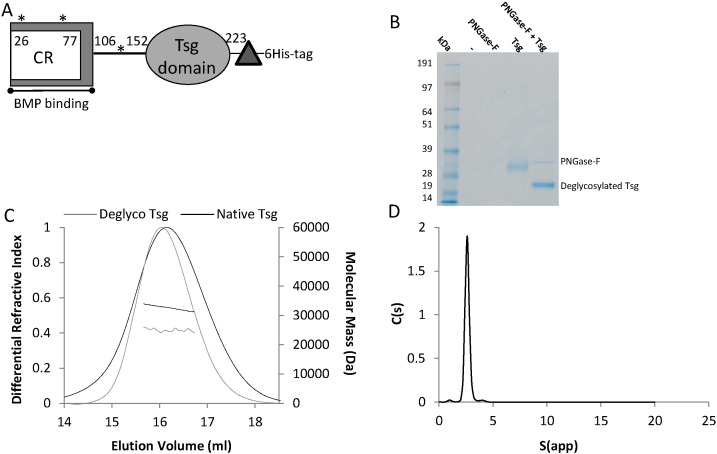
Glycosylation and oligomeric state of Tsg. (A) Schematic diagram of Tsg. Tsg has previously been predicted to be composed of two domains, the first having homology to the cysteine-rich (CR) von Willebrand C-type domains in chordin and binds to BMPs [14] whereas the second (Tsg-specific domain of as yet unknown function) also contacts a high number of cysteine residues but has no known homologues. The domains are separated by a putative hinge region and both domains appear to be required for chordin binding [38]. The construct contains a 6-His-Tag for purification and a thrombin cleavage site for tag removal (triangle). Potential N-linked glycosylation sites indicated by asterisks. (B) Coomassie stained SDS-PAGE gel showing that purified Tsg can be deglycosylated under non-denaturing conditions. (C) MALS profile of native and deglycosylated Tsg. Refractive index and calculated molecular weight against elution volume from the size exclusion column (ml) showing the shift in elution volume and size following deglycosylation. Native Tsg (black line) has a molecular mass of 32.3 kDa (± 0.13%), ~ 30% larger than deglycosylated Tsg (grey line) (25.0 kDa (± 0.5%)) experimental errors from polydispersity. In both states the mass of Tsg is consistent with a monomer in solution (theoretical mass based on peptide sequence is 23.8 kDa). (D) Velocity AUC data for native Tsg showing a single species with mass ~ 36.7 kDa, S_20_W of 2.57, hydrodynamic radius of 3.44 nm and frictional ratio of 1.27.

Tsg has previously been predicted based on sequence analysis to be a two-domain protein and is suggested to consist of an N-terminal cysteine-rich domain homologous to a vWC domain [14] and a C-terminal domain separated by a putative hinge region [18] (Fig. 1A). However, there is currently no structural evidence to support this prediction and the number and arrangement of cysteine residues in Tsg are not consistent with a vWC domain fold. This paper investigates the oligomeric state and shape of human Tsg using small-angle X-ray scattering (SAXS), multi-angle light scattering (MALS) and analytical ultracentrifugation (AUC). In order to better understand the mechanism of Tsg in mediating interactions between chordin and BMPs, we used surface plasmon resonance (SPR) to determine binding locations. We also used SPR to test for direct interaction between Tsg and mammalian tolloid (mTld). To determine the extent of any direct anti-BMP-4 or -7 activity by Tsg, we used BMP activity assays including inhibition of alkaline phosphatase production, SMAD phosphorylation and BMP-induced gene expression. Moreover we show that Tsg interacts with partially cleaved chordin to increase BMP inhibition and promote further cleavage by tolloids. These findings support the physiological relevance of partially cleaved chordin as an important BMP regulator [19]. Our results point to a monomeric Tsg interacting directly with chordin or BMP and suggest that Tsg has a type-specific regulation of BMPs enabling finely controlled regulation of BMP signalling in conjunction with other regulators.

## Results

### Expression and oligomeric state of twisted gastrulation

Human Tsg was expressed in a mammalian expression system and purified as a secreted protein (Fig. 1B). Tsg is frequently assumed to form a homo-dimer as immunoprecipitation studies have suggested that two molecules of Tsg bind to BMP-4 [14], [15]. Therefore in order to determine the size and oligomeric status of Tsg, multi-angle light scattering (MALS) was used in conjunction with size exclusion chromatography. The MALS profile (Fig. 1C) showed a single species that had a molecular mass of 32.3 kDa ± 0.13%, which is larger than the predicted mass of a monomer (~ 23.8 kDa). However human Tsg has three predicted N-linked glycosylation sites and the glycosylation patterns of Tsg have previously been shown to have functional importance in BMP-binding [9]. Fig. 1B shows that Tsg can be deglycosylated by PNGase-F treatment in non-denaturing conditions. The difference in mobility on SDS-PAGE (*i.e.* molecular mass) following removal of the N-linked sugars appears to be considerable, especially in relation to the size of the protein. Therefore MALS was used to quantify the mass of the protein following deglycosylation. Fig. 1C shows the comparison of molecular mass for deglycosylated and native Tsg of 25.0 kDa and 32.3 kDa, respectively. The MALS data also show that in either the presence or absence of N-linked glycosylation Tsg is monomeric. We confirmed this finding by velocity sedimentation analytical ultracentrifugation (AUC) measurements showing a single species for native Tsg consistent with a glycosylated monomer of 36.7 kDa (Fig. 1D). The hydrodynamic radius of Tsg from MALS was 3.4 nm which is in agreement with the AUC data (3.44 nm) shown in Fig. 1D. The S_20_W was 2.57 S and the frictional ratio 1.27, indicative of a globular protein.

### Nanostructure of Tsg

**Fig. 2:**
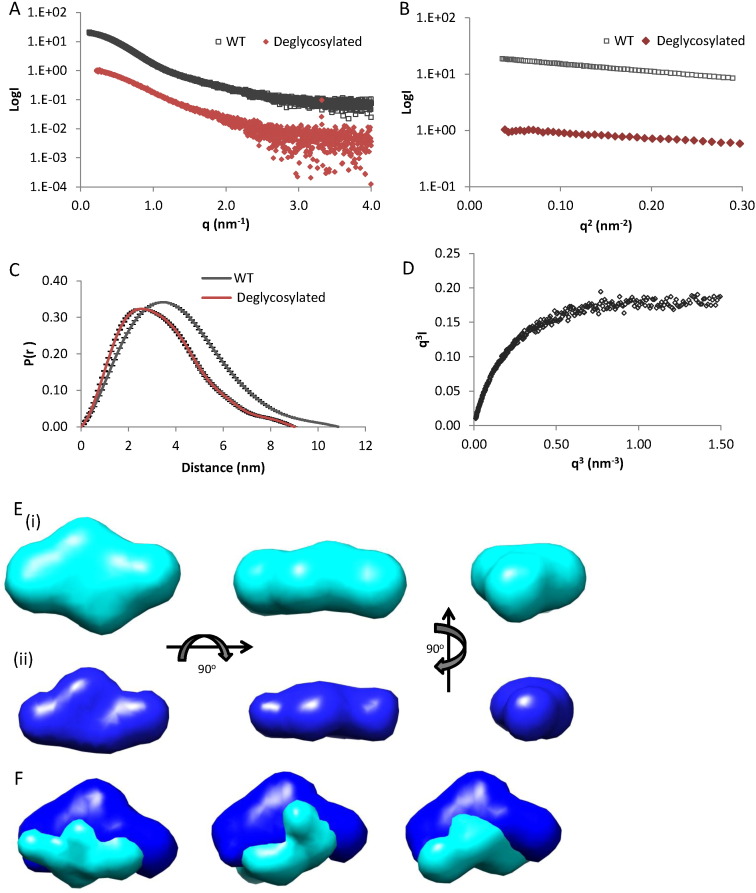
SAXS data on native and deglycosylated Tsg. (A) The experimental SAXS data for the native (black) and deglycosylated (brown) Tsg are plotted as a function of q. (B) The low-angle region of the X-ray scattering data is show in the form of a Guinier plot, which is linear for values q ≤ 1/R_g_. From the Guinier plot, the Rg can be estimated as 3.1 nm for native and 2.5 nm for deglycosylated Tsg. (C) The distance distribution functions for native (black) and deglycosylated (brown) Tsg are shown. The maximum dimensions calculated with Gnom are 11 nm (native) and 9 nm (deglycosylated). (D) The q^3^ plot has a linear plateau which indicates some flexibility in Tsg [22]. (E) Shapes were simulated *ab initio* by the programme DAMMIN [21]. Models for native (cyan (i)) and deglycosylated Tsg (blue (ii)) are each shown in 3 orthogonal views. (F) Modelling of the protein and glycan density was performed with MONSA [21]. The protein density was aligned and averaged and shown in blue, the additional density from the glycans is shown in cyan. Three representative models are shown.

The 3D structures of both deglycosylated and native Tsg in solution were investigated using small-angle X-ray scattering (SAXS) (Fig. 2A). SAXS measurements were made at both Diamond Light Source (I22) and PETRAIII (P12) synchrotron radiation sources. The SAXS data quality was assessed using Guinier plots, to check for aggregation in the sample (Fig. 2B). The radii of gyration (Rg) obtained from the Guinier approximation were 2.5 nm and 3.1 nm for the deglycosylated and native Tsg, respectively. The radius of gyration is consistent with the hydrodynamic radius (3.4 nm from MALS and AUC for native Tsg) which supports the finding that Tsg is monomeric in solution even at high concentrations used for SAXS data collection (6 mg/ml). The maximum particle dimension for native Tsg was estimated as 11 nm using indirect Fourier transform with GNOM [20] (Fig. 2C) corresponding to the longest end-to-end distance in the protein. The maximum dimension for deglycosylated Tsg was lower (9 nm) than for native Tsg as a result of the larger hydration shell attracted by sugar groups. For native and deglycosylated Tsg, *ab initio* models were generated using DAMMIN [21]. The modelling allowed us to fit the experimental data with mean discrepancy factors (*x*) of 0.93 (native) and 1.09 (deglycosylated) and at least 20 simulations for each dataset were completed to determine the common structural features. The mean normalized spatial discrepancy factor between the solutions was 0.52 (native) and 0.60 (deglycosylated) indicating unique solutions. Fig. 2E shows a flattened cross shape for Tsg with dimensions 11 nm × 4.2 nm × 6.3 nm. Two distinct lobes of the protein can be distinguished in the model but the structure is relatively compact and appears to lack a distinct linker that was predicted to exist between domains [18]. However, the q^3^ plot (Fig. 2D) suggests that there is flexibility in Tsg which may indicate that there is movement between the two lobes [22]. The superimposed *ab initio* models of deglycosylated and native Tsg show that the two models have a similar shape with additional density in the native structure close to the centre of the molecule where the sugars may be located (Fig. 2F).

### Secondary structure and domain analysis

**Fig. 3:**
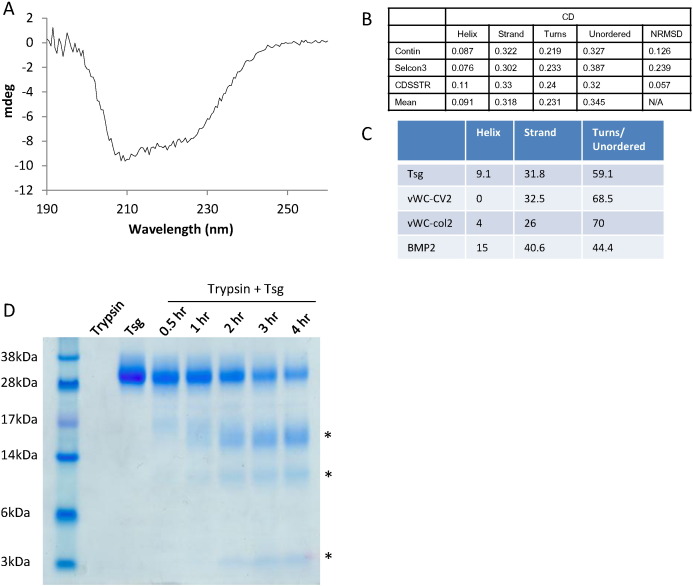
Circular dichroism and secondary structure of Tsg. (A) Circular dichroism spectrum for 0.4 mg ml^− 1^ native Tsg showing mdeg against wavelength. The graph shape is characteristic of a mix of α-helix and β-sheet secondary structure. (B) Table summarizing analysis of CD data for Tsg using the Contin, Selcon3, CDSSTR and K2d algorithms. Mean values exclude K2d owing to high normalized root-mean-square deviation (NRMSD) values. (C) Comparison of predicted secondary structure values for Tsg with secondary structure of known vWC domain structures and cysteine knot fold. (D) Limited proteolysis of Tsg with trypsin (1:50 *w*/w). After 30 min cleavage of Tsg was observed, leading to the appearance of 3 bands (marked by asterisks).

The structure of Tsg is currently unknown but it has been suggested that the cysteine-rich region has homology to a von Willebrand Factor-C domain [14]. To determine whether the secondary structure composition of Tsg is similar to a vWC domain, circular dichroism spectra were collected on native Tsg (Fig. 3A). The CD profile has a characteristic double dip with a deeper trough at 208 nm typical of α/β secondary structure. Deconvolution of the spectra predicted ~ 9% α-helix and ~ 32% β-sheet shown in Fig. 3B which is compared to the secondary structure content of vWC domains from CV-2 [23] and collagen IIa [24] as well as a cysteine knot protein (BMP-2) [23]. The CD data suggests that Tsg has a greater α-helical content than a single vWC domain and less coil/unordered regions which indicates Tsg may have more extensive secondary structural elements than a vWC domain (Fig. 3C). In order to determine whether Tsg has two domains separated by a flexible linker, limited proteolysis with trypsin was performed. After 30 min, some Tsg had been cleaved and could be separated into three prominent bands on SDS-PAGE. These species have molecular weights of approximately 16, 10 and 3 kDa (Fig. 3D). These sizes are consistent with cleavage at two potential trypsin cleavage sites in the putative linker. These data suggest that this linker sequence is accessible to trypsin and there are two main species following cleavage. However, only a proportion of Tsg is initially cleaved which indicates that there is some protection of this region in the folded protein.

### A high-affinity Tsg binding site is localized towards the C-terminus of chordin

**Fig. 4:**
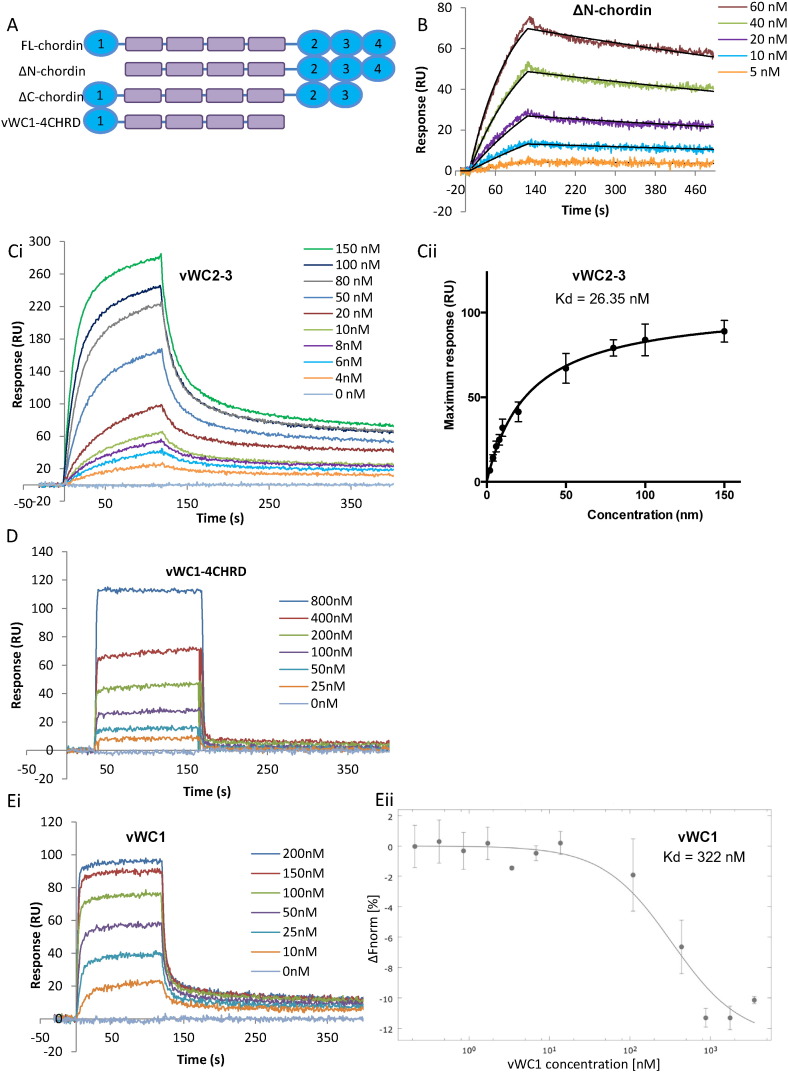
SPR sensorgrams showing Tsg binding to chordin constructs (A) Schematic diagram of chordin constructs used in this analysis. Tsg was immobilised on a Proteon GLC chip *via* amine coupling. ΔN-chordin and vWC2–3were injected on to Tsg (300 RUs immobilised) at increasing concentrations. (B) ΔN-chordin fitted 1:1 Langmuir binding model shown in black. (C) The binding affinity for vWC2–3 was determined using equilibrium analysis. (D) vWC1-4CHRD did not bind to Tsg and vWC1 bound weakly in a highly biphasic manner (Ei) (for (D) and (Ei) 800 RUs of Tsg were immobilised). MST was performed to detect in solution binding between Tsg and vWC1. Tsg was labelled and added to increasing concentrations of vWC1, a K_D_ of 322 nM was determined (Eii). All experiments were performed in triplicate.

A previous study had shown that individual chordin domains bound Tsg with much lower affinity than the full length chordin molecule and suggested that vWC1, 3 and 4 all bound Tsg weakly [25]. We screened different fragments of chordin for Tsg binding using surface plasmon resonance (SPR) to determine which domains underpin higher affinity binding. Chordin constructs containing the C-terminal vWC domains (Fig. 4A) were able to bind to immobilised Tsg with high affinity (Fig. 4B and C; ΔN-chordin (K_D_ = 3.08 nM) and vWC2–3 (K_D_ = 26.35 nM)). The interaction between ΔN-chordin and Tsg fits a simple 1:1 Langmuir binding model, however the interaction between Tsg and vWC2–3 deviates from this fitting, especially at higher analyte concentrations therefore the dissociation constant was derived from equilibrium analysis (Fig. 3 Cii). The central region of chordin (vWC1-4CHRD region) does not bind to Tsg under these experimental conditions (Fig. 4D). The N-terminal BMP-binding domain (vWC1) bound in a highly biphasic manner, with large amounts binding but a very rapid dissociation (Fig. 4 Ei). It appears that there is an initial vWC1–Tsg interaction then a secondary interaction, which could be vWC1 self-association or binding of vWC1 to a second site on Tsg. This interaction was also analysed in solution with microscale thermophoresis (MST) and a K_D_ of 322 nM determined (Fig. 4 Eii). Together these data suggest that high-affinity binding of Tsg to chordin occurs *via* the C-terminal vWC2–3 domains consistent with immunoprecipitation data from Larrain and co-workers [15], a second weaker interaction site is present on vWC1 consistent with previous binding studies [25], [26] which appears more accessible following cleavage from the 4CHRD region. Natively deglycosylated Tsg still bound to full-length chordin with high affinity so the N-linked glycans do not appear to play a part in this interaction (Supplementary Fig. 1).

### Tsg inhibition of BMP signalling

**Fig. 5:**
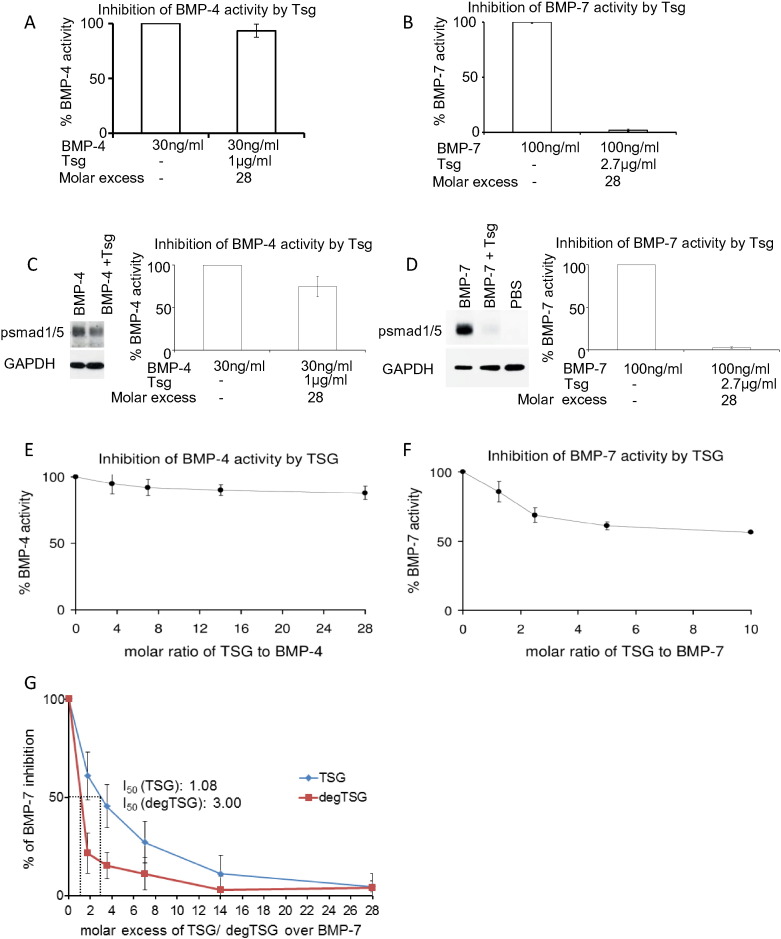
Bioactivity of Tsg for BMP-4 and-7. Inhibition of BMP-4 and BMP-7 growth factor activity by Tsg was detected by inhibition of alkaline phosphatase production in C2C12 cells (A and B) and Smad-1/− 5 phosphorylation in HEK293T cells (C and D) with a 28 × molar excess of Tsg. A rapid qPCR assay based on the *Id3* expression in C2C12 cells after 5 h of incubation with growth factor was also used. A range of Tsg concentrations were tested with BMP-4 (E) and BMP-7 growth factor (F). (G) Natively deglycosylated Tsg was still able to inhibit BMP-7 as detected by inhibition of alkaline phosphatase production. Errors represent standard deviation from at least three independent experiments.

In order to investigate whether Tsg had an effect on BMP signalling in the absence of chordin, BMP-inhibition assays were performed. The inhibition of BMP-induced alkaline phosphatase production (Fig. 5A and B) and phosphorylation of Smad-1/− 5 (Fig. 5C and D) by an excess of Tsg was analysed. Tsg was an ineffective BMP-4 inhibitor but showed complete inhibition of BMP-7 signalling. Using a qPCR based activity assay to determine the effect on BMP target gene expression, a range of Tsg:BMP molar ratios were screened and showed that little additional BMP inhibition was observed when increasing the molar ratio of Tsg:BMP-7 above 2 monomers:1 dimer (Fig. 5F). Native deglycosylation did not affect binding between BMP-7 and Tsg (Supplementary Fig. 1) and deglycosylated Tsg was a more effective inhibitor of BMP-7 (Fig. 5G).

### Tsg interaction with partially cleaved chordin

**Fig. 6:**
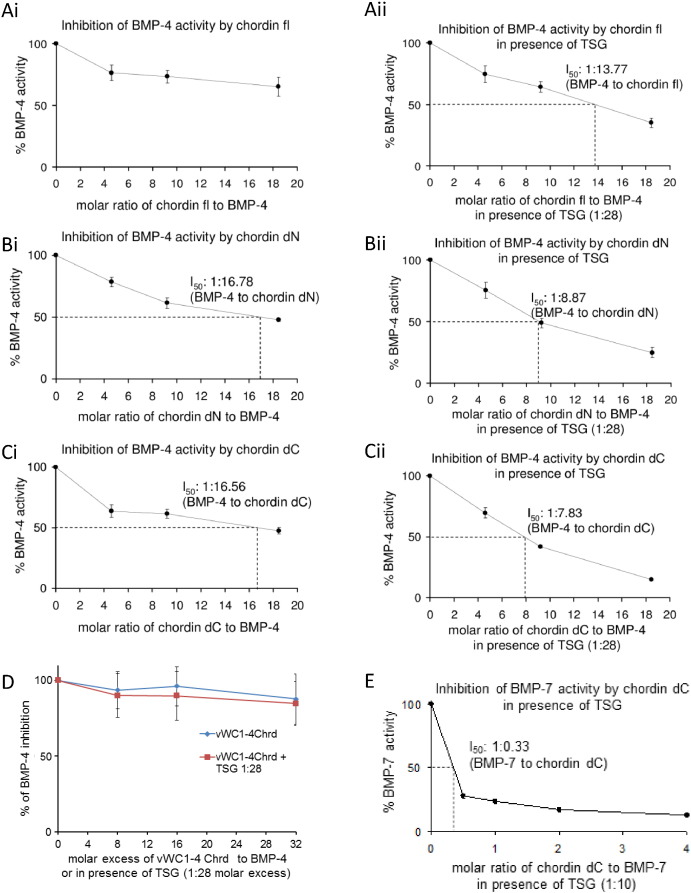
Bioactivity of chordin and chordin fragments enhanced by Tsg (A) Inhibition of BMP-4 growth factor activity by chordin was detected by *Id3* reporter assay in the presence or absence of Tsg. Tsg increases inhibition of BMP-4 by both full length chordin and the chordin cleavage fragments (B) ∆N- and (C) ∆C-chordin. (D) vWC1-4CHRD only weakly inhibits BMP-4 and this is not enhanced by Tsg. (E) BMP-7 activity was inhibited by increasing amounts of ∆C-chordin in the presence of Tsg. Errors represent standard deviation from three independent experiments.

Tsg increases the inhibitory capacity of full length chordin, but competes with fully cleaved chordin for BMP binding. What is not clear is how Tsg interacts with partially cleaved chordin fragments; therefore as BMP-4 was not inhibited directly by Tsg, the effect of Tsg on BMP-4 inhibition by chordin was assessed. Fig. 6A shows that as expected Tsg increases the BMP-4 inhibitory effect of FL-chordin. Fig. 6B and C show that Tsg also enhances inhibition by ∆N- and ∆C-chordin, respectively. The observed effect must be mediated through chordin as Tsg did not inhibit BMP-4 directly (Fig. 5) and shows that Tsg increases the capacity for BMP inhibition of full length chordin and the larger cleavage fragments. In contrast, the shorter vWC1-4CHRD fragment only inhibited BMP-4 signalling weakly and Tsg was ineffective at enhancing inhibition (Fig. 6D) which is consistent with the weaker binding seen between Tsg and vWC1. BMP-7 activity was inhibited effectively by increasing amounts ∆C-chordin with a 1:10 ratio of Tsg where only small amounts of ΔC-chordin were required to completely inhibit BMP-7 (Fig. 6E).

### Tsg does not interact directly with mTld

**Fig. 7:**
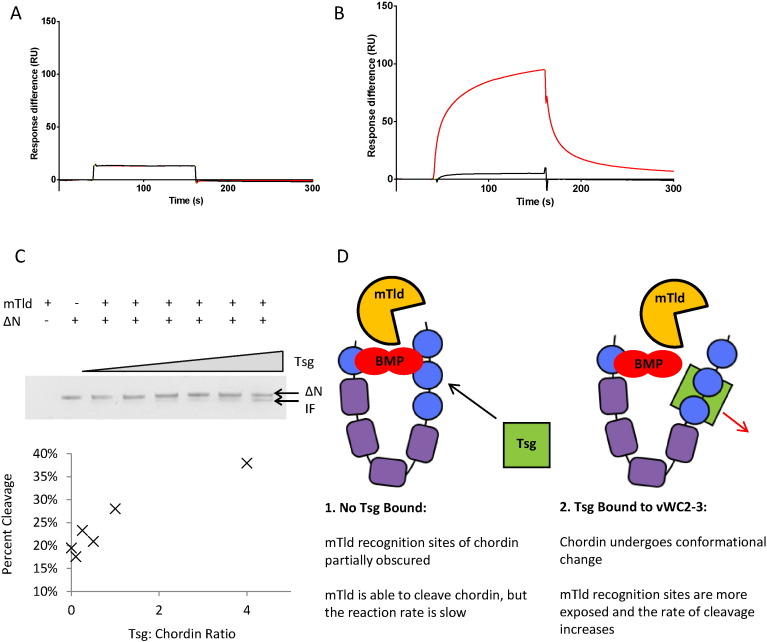
SPR sensorgrams showing mTlD does not bind directly to Tsg. Immobilised proteins were cross-linked to a CM5 chip *via* amine coupling. 830 RU of Tsg was immobilised on the chip and two analytes were injected onto the immobilised Tsg: 500 nM mTlD (black response curve) and 500 nM mTlDC4C5 (red response curve). (B) Two analytes were injected onto the immobilised mTlD (2400 RU): 500 nM Tsg (black response curve) and 500 nM ΔN-chordin (red response curve). (C) ∆N-chordin cleavage assay by mTld in the presence of increasing amounts of Tsg (0:1, 0.1:1 0.25:1, 0.5:1, 1:1 and 4:1 molar ratio of Tsg:∆N). The emergence of the chordin cleavage product, intermediate fragment (IF) is labelled. A plot of the percentage of cleaved chordin against Tsg:chordin ratio is shown. (D) Schematic diagram demonstrating proposed mechanism of conformational change in chordin following Tsg binding exposing tolloid recognition sites.

Tsg increases the rate of chordin cleavage by tolloids but the mechanism for this is not clear. Given that this effect is also observed *in vitro* when Tsg, mTld and chordin are isolated from other pathway components, the two possibilities are that either Tsg binds to tolloid and chordin simultaneously acting as a scaffolding protein or Tsg induces a conformational change in chordin making it more accessible to tolloid cleavage. To investigate the former, we determined whether there was an interaction between Tsg and mTld using SPR (Fig. 7A). The interaction of mTld with Tsg as both ligand and analyte was screened, however no binding was detected in either configuration (Fig. 7A and B). In addition, the C-terminal domains of mTld were also screened for binding as these domains are known to have the highest affinity interaction with tolloid substrates [27] but these domains showed no interaction with Tsg either (Fig. 7A) whereas on the same chip ∆ N-chordin bound to mTld (Fig. 7B). This suggests that Tsg does not act as a scaffold between chordin and mTld and provides further support for a conformational change in chordin following Tsg binding. In order to demonstrate that Tsg is able to enhance mTld cleavage of chordin in the absence of other factors *e.g.* BMPs, ∆N-chordin was incubated with mTld in the presence of increasing concentrations of Tsg showing a dose dependent increase in rate of cleavage in the presence of Tsg (Fig. 7C). Fig. 7D shows a schematic diagram of the proposed interaction.

## Discussion

Using small angle X-ray scattering we show the nanoscale structure of Tsg, an elongated globular-like molecule and is not, as previously postulated a dimer [14], [15] but rather a stable monomer in solution. Although, it is possible that two monomers of Tsg may interact with a BMP dimer as cross-linking studies have detected a 2:2 complex of *Xenopus* Tsg-BMP-4 [15]. A relatively high proportion of the molecular weight of Tsg is contributed by N-linked glycans. This property is likely to make Tsg highly soluble and stable in the matrix, supporting the hypothesis that it plays a role in stabilizing BMP and facilitates diffusion of Tsg containing complexes. In agreement with that is a study showing that the glycosylation patterns in human Tsg and its homologues play a key role in BMP binding specificity [9]. Our structure shows that the sugars are surface-accessible and located between the two lobes of the protein. The structure of Tsg has an elongated flat face where glycosylation adds a bulge to this surface and could provide multiple binding sites for BMPs and thereby supporting its role as a BMP solubilising factor [28].

Our SPR solid phase binding experiments showed Tsg bound with high affinity to the C-terminal vWC2–3 region of chordin and this binding was unaffected by natively deglycosylating Tsg. Tsg bound weakly to the vWC1 domain alone but not in the presence of the adjacent 4CHRD region which is consistent with previous studies where mouse Tsg has been shown by immunoprecipitation to bind to the vWC1 domain of mouse chordin [6] and weakly by SPR (K_D_ = 0.2–0.9 μM) [25], [26]. We were unable to detect any direct binding using SPR between mammalian tolloid or tolloid domains and Tsg, despite both binding to chordin and Tsg enhancing tolloid cleavage of chordin in the absence of other factors. This is consistent with other studies that did not detect a direct interaction by immunoprecipitation [6]. If Tsg were acting as a scaffold between chordin and tolloid, any interaction would be expected to be strong and relatively stable. This finding, combined with the ability of Tsg to introduce new cleavage sites in some species [6], is a strong indicator that the mechanism for enhancing chordin cleavage by tolloids is through inducing a conformational change in chordin. This could subsequently enhance tolloid cleavage of chordin perhaps by making cleavage sites more readily available or presenting a cryptic cleavage site. This mechanism seems more efficient because it excludes a scenario in which excess Tsg remains bound to tolloid while it processes other targets unrelated to the chordin-BMP pathway.

Tsg acts to strengthen the inhibitory complex between BMP and chordin but we also found that in the absence of chordin, Tsg has significant BMP-7 inhibitory capacity. However this was not the case for BMP-4. It has previously been demonstrated that Tsg has a weak inhibition of BMP-2, and could inhibit the BMP-2/BMPR-IA interaction but not BMP-2/ActR-IIB binding [25]. Together these findings raise the possibility of differential Tsg interaction and mechanism depending on the specific BMP type. It has previously been shown that Tsg and chordin mask different BMPR-binding sites on BMPs [25]. Taken together this suggests that, in addition to strengthening the ternary complex, Tsg also increases inhibition of BMP-7 by blocking binding sites which chordin cannot.

Our previous findings showed that partially cleaved chordin retains BMP inhibitory activity at similar or higher levels to full length chordin and may represent a mechanism to modulate BMP inhibition by chordin [13]. Our new data extend this mechanism, where the presence of Tsg allows differentiation between and selective inhibition of BMPs. We postulate that in the absence of Tsg, chordin cleavage resulting in ΔN-chordin is a way to abolish chordin's inhibitory capacity for BMP-7 whereas chordin cleavage resulting in ΔC-chordin may represent an alternative way to inhibit BMP activity more efficiently [13]. This effect is less pronounced for BMP-4 than for BMP-7 (comparing Fig. 6A-C to Fig. S7 from [13]). This mechanism is probably needed in the absence of Tsg as in the presence of Tsg, full-length, ΔC- and ΔN-chordin all show a similarly enhanced inhibition rate (50% inhibition at a molar excess of 1:8–13 over BMP-4). However in contrast to BMP-4, Tsg is inhibited directly by BMP-7 (Fig. 5) so chordin may not be required when Tsg is present. However, ΔC-chordin and Tsg show the best inhibition rate which could be additive. With this mechanism it is possible, for example, in the presence of Tsg to block BMP-7 activity completely while BMP-4 is still active. For instance, at a 1:10 ratio of Tsg, only small amounts of ΔC-chordin are necessary to completely inhibit BMP-7 (Fig. 6E) while BMP-4 activity is not affected at these concentrations (Fig. 6 Cii). Native deglycosylation of Tsg even enhances BMP-7 inhibition which adds another layer of tissue-specific modulation. This in contrast to non-glycosylated Tsg expressed in bacteria that had reduced binding to BMP-2 and reduced activity in a mandibular explant culture system [9]. This may indicate further differences between BMP sub-types or that natively deglycosylated Tsg retains some glycans.

We show that Tsg increases inhibition of BMPs by chordin fragments, while promoting their further cleavage by mTld. This supports the requirement of dual cleavage of chordin for activation of BMP signalling. In addition chordin is expressed as a number of tissue specific splice variants [29] and our data suggest that their regulation by Tsg is likely to be similar to full length chordin, provided they retain the Tsg-binding vWC2–3 domains. In summary, we show a nanoscale structure for monomeric Tsg supported by MALS and AUC data, we propose that Tsg acts primarily as a BMP agonist by inducing conformational change in chordin to make it more accessible for cleavage by tolloids. Tsg increases BMP inhibition with both full length chordin and partially cleaved chordin, which suggests chordin continues to inhibit BMPs until complete cleavage has occurred.

## Experimental procedures

### Protein expression and purification

Human Tsg was generated by PCR from image clone Q9GZX9 (Bioscience Gene Service). The chordin vWC2–3 (residues 701–860), vWC1 (residues 50–128) and vWC1-4CHRD (residues 27–650) constructs were generated by PCR from full-length human chordin. His_6_ tags were incorporated at the C-termini following a thrombin cleavage site. Constructs were ligated into a modified pCEP4 vector [30] and transfected into HEK 293-EBNA cells cultured as described previously [13]. Proteins were purified by nickel affinity chromatography and size-exclusion chromatography on an AKTA purifier FPLC using a Superdex 200 10/300GL column (GE Healthcare). Protein identity was confirmed by in-gel trypsin digestion and LC–MS/MS using a NanoAcquity LC (Waters) coupled to a LTQ Velos (Thermo Fisher Scientific). Additional human chordin constructs (ΔN- and ΔC-chordin) and mammalian tolloid were also expressed in HEK 293-EBNA cells as previously described [13], [31]. Except where otherwise stated buffer conditions for all experiments were 10 mM Tris–HCl, 150 mM NaCl, pH 7.4. Protein concentration was estimated using absorbance at 280 nm corrected for the molar extinction coefficient.

### Native deglycosylation and limited proteolysis

Tsg was digested in 0.04 U/ml of PNGase-F at 37 °C for 36 h. Removal of the sugars was verified using SDS-PAGE and the digested protein separated from the enzyme using size exclusion chromatography. Proteolysis with trypsin (1:50, *w*/w) was carried out at 37 °C. At the times indicated, samples were withdrawn, and the reaction was stopped by the addition of 2 × SDS sample buffer containing 100 mm dithiothreitol and heating at 95 °C for 4 min as described in [32].

### Multi-angle light scattering

Tsg samples (0.5 ml at approximately 0.5 mg/ml) were loaded onto a Superdex 200 10/300GL column running at a flow rate of 0.5 ml/min. Proteins eluting from the column passed through a Wyatt DAWN Heleos II EOS 18 angle laser photometer with QELS detector (Wyatt Technologies) and an Optilab T-rEX refractive index detector. The molecular mass and concentrations of the resulting peaks were analysed using ASTRA 5.6.

### Velocity sedimentation analytical ultracentrifugation

Velocity sedimentation AUC was performed on Tsg (~ 0.2 mg/ml) at 25,000 rpm using a Beckman XL-A analytical ultracentrifuge with an An60Ti 4-hole rotor at 20 °C. The sedimenting boundary was monitored at 230 nm every 90 s for 300 scans with a radial step size of 3 μm. Data were analysed using Sedfit [33] and the resulting apparent sedimentation coefficient was corrected for standard conditions using the programme SEDNTERP [34]. The hydrodynamic radius (R_h_) and frictional ratio (*f*/*f*_*o*_), which represents the deviation of the friction of the molecule from a theoretical sphere of the same molecular weight, were also calculated using SEDNTERP.

### Small-angle X-ray scattering

SAXS data were collected at 1–6 mg/ml on native and deglycosylated Tsg at the Diamond Light Source (I22) and PETRA III (P12) beamlines. Samples were maintained at 10 °C during exposure using the standard sample holders at each beamline. Data was checked for radiation damage between subsequent frames and different concentrations merged using PRIMUS [35]. The forward scattering intensity, Rg and one-dimensional intra-particle distance distribution function P(r) in real space were evaluated with the indirect Fourier transform programme GNOM [20] and particle shapes were restored *ab initio* using DAMMIN [21]. At least 20 *ab initio* models were calculated with each programme these were combined into a single model using DAMAVER suite [36]. Multiphase volumetric modelling was performed using MONSA [21] to analyse the difference in density between the native and deglycosylated Tsg data. Models were generated with MONSA online using a phase contrast of 1.6 for the glycans. 10 models were calculated then the volume corresponding to Tsg was aligned and averaged using the methods described in [37].

### Circular dichroism

Circular dichroism measurements were performed on 0.4 mg/ml Tsg using a Jasco J715 spectropolarimeter at 190–260 nm and 0.05 cm path length using the sample buffer as a reference. The mean of twenty measurements was taken at 100 mdeg sensitivity at a rate of 20 nm/min. The estimation of protein secondary-structure content was analysed using Contin-LL, Selcon 3, CDSSTR and K2d programmes (all available algorithms provided on the DICHROWEB server www.dichroweb.cryst.bbk.ac.uk). The results of each algorithm were averaged to obtain a quantitative analysis of the secondary structural elements.

### Surface plasmon resonance

Binding analyses were performed using either a Biacore T200 (GE Healthcare, Little Chalfont, UK) with a Series S CM5 sensor chip (GE Healthcare) or a ProteOn XPR36 (Bio-Rad Laboratories Ltd., Hemel Hempstead, UK) with a ProteOn GLC sensor chip (Bio-Rad). Ligands were immobilised on the sensor chips (concentrations ranging from 3 to 9 μg/ml in 50 mM sodium acetate pH 4.0) *via* amine-coupling using an EDC/NHS chemical cross-linker following the manufacturer's instructions and subsequently blocked with ethanolamine. The native Tsg interactions in Fig. 4 were examined using the ProteOn XPR36 at 25 °C in 10 mM HEPES, 150 mM NaCl, 3 mM EDTA, 0.05% Tween-20, pH 7.4 with a flow rate of 50 μl/min. All other interactions were examined using the Biacore T200 at 25 °C in 10 mM Hepes, 150 mM NaCl, 3 mM EDTA, 0.005% Tween-20, pH 7.4 with a flow rate of 30 μl/min. For kinetic analysis, analytes were injected at several concentrations onto immobilised ligands. Kinetic constants were calculated by nonlinear fitting of kinetic models (including 1:1 binding and heterogeneous analyte) to the experimental data (the recorded association and dissociation curves) using the ProteOn Manager. Calculated kinetic constants included the association rate constant (k_a_), the dissociation rate constant (k_d_) and the equilibrium dissociation rate constant (K_D_). K_D_ was derived from the ratio of k_d_ to k_a_.

### Microscale thermophoresis

Tsg was labelled using the Monolith NT Protein labelling Kit Red-NHS kit from NanoTemper Technologies. Unbound fluorophore was removed using a desalting column equilibrated with MST buffer (50 mM Tris, 150 mM NaCl, 10 mM MgCl_2_ 0.05% Tween-20 pH 7.4). 1:1 serial dilutions of vWC1 at a starting concentration of 7 μM in MST buffer were mixed 1:1 with 15 nM NT647-labelled Tsg and loaded into standard treated capillaries and analysed using the Monolith NT.115Pico from NanoTemper Technologies at 20% LED power and 20% MST power. The experiment was done in triplicate and the K_D_ determined.

### Alkaline phosphatase assay

Dose response curves for BMP-4 and BMP-7 induced ALP production in C2C12 cells were generated to determine the ED50 for BMP-4 (27 ng/ml (1.0 nM)) and BMP-7 (100 ng/ml (3.2 nM)) (Supplementary Fig. 2). C2C12 cells were seeded into 96-well plates (Costar) at a density of 30,000 cells/well in DMEM (MediaTech) containing 2% *v*/v FCS. BMP stimulation was carried out in triplicates with 30 ng/ml BMP-4, or 100 ng/ml BMP-7 (both human from R&D Systems) in the presence or absence of 28-fold molar excess of Tsg for 72 h. Prior to stimulation proteins were dialyzed together in PBS, containing 0.01% BSA as carrier. After stimulation, cell layers were washed twice with PBS and lysed with 100 μl lysis buffer (0.1 M glycine pH 9.6, 1 mM MgCl_2_, 1 mM ZnCl_2_) containing 1% NP-40. Lysates were clarified by centrifugation at 18,800 *g*. 100 μl Alkaline Phosphatase Yellow (pNPP) Liquid Substrate (Sigma-Aldrich) were added to 50 μl of each lysate and incubated for 20 min at room temperature followed by measurement of activity in a TECAN infinite M1000 reader (Dynamic Instruments, Wilmington, DE) at 405 nm.

### Phospho-smad Western blot assay

HEK 293T cells were seeded into 6-well plates at a density of 1000,000 cells/well in DMEM. The following day, cells were stimulated for 1 h with 30 ng/ml BMP-4 or 100 ng/ml BMP-7 in the presence or absence of 28-fold molar excess of Tsg. After stimulation cell layers were incubated with lysis buffer (10 mM NaCl, 1.5 mM MgCl_2_, 20 mM HEPES pH 7.4, 20% glycerol, 0.1% triton X-100, and 1 mM DTT), centrifuged at 2000 rpm for 5 min at 4 °C, and washed with PBS. Aliquots of the supernatant were supplemented with 1 × PhosSTOP (Roche) and western blotted. Phosphorylated Smad was detected using pooled anti-phosphosmad1 (1:1250) and Smad5 (1:1000) antibodies (Abcam). Band intensities were quantitated with ImageJ and plotted against concentration.

### BMP-induced expression of Id3

C2C12 cells were seeded into 96-well plates at a density of 30,000 cells/well in DMEM without serum. Proteins were dialyzed together in PBS, 0.01% BSA overnight. BMP stimulation was carried out in the presence of 30 ng/ml BMP-4, or 100 ng/ml BMP-7 for 5 h. After incubation, total RNA from three wells was harvested using TRIzol® reagent (Invitrogen). Total RNA preparations were quantified by photospectrometry. 0.1 μg of RNA per sample was reverse-transcribed using the Bio-Rad iScript™ cDNA synthesis kit. Reverse-transcribed samples were amplified in triplicates with primers for *Id3*, a BMP-responsive element, using the iTaq™ SYBR Green Supermix (Bio-Rad). Analysis of data was performed using the 2^− ΔΔ*Ct*^ method and quantitated relative to the *Arbp PO* gene. Gene expression was normalized to samples where cells were incubated with corresponding amounts of BSA, which provided an arbitrary constant for comparative fold expression.

### mTld cleavage assay

50 μg ml^− 1^ ∆N-chordin was incubated with 3 μg ml^− 1^ mTld at 37 °C for 0.5 h. Tsg was added in a 0.1:1, 0.25:1, 0.5:1, 1:1 and 4:1 molar ratio of Tsg:∆N-chordin. At 1:1 or higher rations of Tsg:∆N-chordin an increase in tolloid cleavage was observed. Gels were digitised and bands quantitated using ImageJ.
