# Supplementary material for: Structural characterization of twisted gastrulation provides insights into opposing functions on the BMP signalling pathway
Source: Matrix Biol. 2016 Sep;55:49–62. doi: 10.1016/j.matbio.2016.01.019 (PMC5080453; doi:10.1016/j.matbio.2016.01.019)
Supplement: Supplementary file 1 — Supplementary figures [file mmc1.pdf]

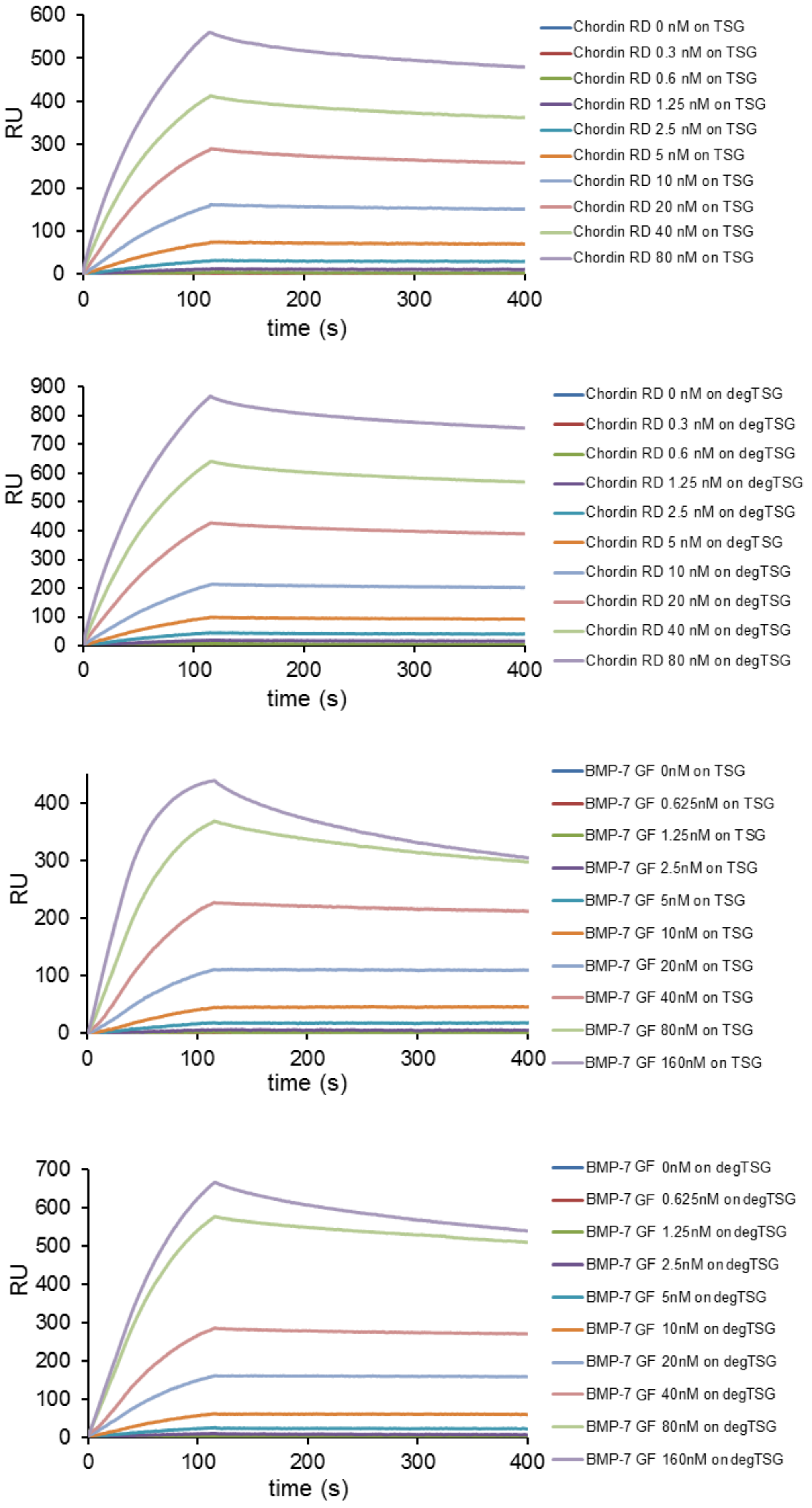

Supplementary Figure 1: SPR sensorgrams showing native and deglycosylated Tsg binding to chordin and BMP-7. Tsg was immobilised on Biacore CM5 chip via amine coupling. Full-length chordin and BMP-7 (both R&D systems) were injected on to either native or deglycosylated Tsg (1200 RUs immobilised) at increasing concentrations.

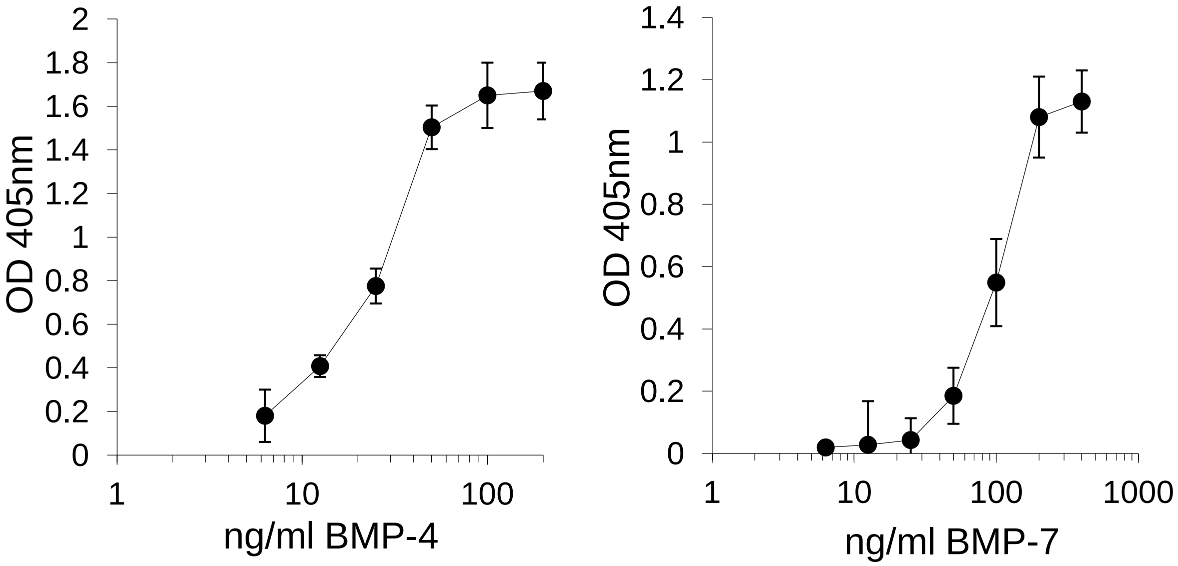

Supplementary Figure 2: Dose response curves of BMP-4 and -7 in ALP assays.
Titration experiments were performed to generate dose response curves for BMP-4 and BMP-7 induced ALP production in C2C12 cells. The ED50 were determined for BMP-4 (27 ng/ml (1.0 nM)) and BMP-7 (100 ng/ml (3.2 nM)).
